# Supplementary material for: Effect of Immature Rubus occidentalis on Postoperative Pain in a Rat Model
Source: Medicina (Kaunas). 2023 Jan 30;59(2):264. doi: 10.3390/medicina59020264 (PMC9958716; doi:10.3390/medicina59020264)
Supplement: Supplementary file 1 [file medicina-59-00264-s001.zip › Supplementary file S2_Statistical analysis detail.pdf]

Statistical analysis

The primary aim of this study was to evaluate the analgesic effect of iROE after plantar incision. Specifically, this was to be achieved by estimating the differences in MWT (using von Frey filaments) between the control, iROE 10 mg/kg, iROE 30 mg/kg, iROE 100 mg/kg, and iROE 300 mg/kg groups (Experiment 1). To estimate the group size for this study, a pilot study was conducted wherein MWT was measured using six incisional-pain-model rats (group C). As the mean MWT determined in the pilot study did not pass the Shapiro-Wilk test, we planned to analyze the data after natural log transformation. The mean natural log–transformed MWTs at baseline, AI, and 15 min, 30 min, 45 min, 60 min, 80 min, 100 min, 120 min, 24 h, 48 h, and 7 days after administration of iROE were 4.41, 0.96, 0.08, 0.03, 0.02, 0.02, 0.06, 0.06, 0.13, 0.68, 1.40, and 2.80 ln (mN), respectively. The standard deviations of the mean natural log–transformed MWTs ranged from 0.01 to 0.53, and there was an autocorrelation of 0.6 between adjacent measurements on the same individual. For our power calculation, we assumed that first-order autocorrelation adequately represents the autocorrelation pattern. To compare between-group differences, we planned to use the Geisser-Greenhouse Corrected F test for repeated-measures analysis of variance (ANOVA). We wanted to detect 30%, 40%, 50%, and 60% increases in MWT in the 10 mg/kg, 30 mg/kg, 100 mg/kg, and 300 mg/kg iROE groups relative to group C. Consequently, the standard deviation was 0.22, and the standard deviation of actual effect was 0.14; therefore, the effect size was 0.77. With an α of 0.05 and a power of 80%, we needed six rats per group. PASS 11 software (NCSS, Kaysville, UT, USA) was used to calculate the sample size.

The Shapiro-Wilk test was used to determine if the data were normally distributed. The data for the IL-1β, IL-6, and TNF-α levels, as well as those from the rotarod experiments, were normally distributed. However, the MWT data were skewed according to the Shapiro-Wilk test results; therefore, natural log transformation was performed, and the natural log–transformed variables passed the Shapiro-Wilk test.

We, therefore, assumed that the normal distribution assumption for parametric testing was not violated and decided to apply repeated-measures ANOVA: within-subjects factor of times (at baseline, AI, 15 min, 30 min, 45 min, 60 min, 80 min, 100 min, 120 min, 24 h, 48 h, and 7 days) and between-subjects factor of groups (between the control, iROE 10 mg/kg, iROE 30 mg/kg, iROE 100 mg/kg, and iROE 300 mg/kg groups, or between the iROE 300 mg/kg and mROE 300 mg/kg groups).

Given that IL-1β, IL-6, and TNF-α passed Mauchly's sphericity test, they were compared using repeated-measures ANOVA followed by the Tukey test. Given that Mauchly's sphericity test indicated that the assumption of sphericity for MWT had been violated, we used one-way Wilk's lambda multivariate analysis of variance (MANOVA) with each group as an independent factor and the MWTs at each time point (at baseline, AI, 15 min, 30 min, 45 min, 60 min, 80 min, 100 min, 120 min, 24 h, 48 h, and 7 days) as dependent variables. To compare MWT at each time point, univariate ANOVA with Bonferroni correction ($\alpha = 0.05/12 = 0.0042$) was used.

When the homoscedasticity requirement using Levene's test for homogeneity of variances was not met in the ANOVA, we used Welch's corrected ANOVA. To identify the groups with statistically significant mean differences, we used Tukey's or Tamhane's T2 post hoc test when ANOVA or Welch's corrected ANOVA was significant. Individual measurements are expressed as the mean ± standard deviation. Statistical analyses were conducted using SPSS Statistics for Windows, version 26.0 (IBM Corp., Armonk, NY, USA). A p-value ≤ 0.05 was considered statistically significant.
